# Immobilization of Naringinase from *Penicillium decumbens* on Chitosan Microspheres for Debittering Grapefruit Juice

**DOI:** 10.3390/molecules24234234

**Published:** 2019-11-21

**Authors:** Joanna Bodakowska-Boczniewicz, Zbigniew Garncarek

**Affiliations:** Department of Biotechnology and Food Analysis, Wroclaw University of Economics and Business, 53-345 Wroclaw, Poland; joanna.bodakowska@ue.wroc.pl

**Keywords:** naringinase, immobilization, chitosan microspheres, grapefruit juice, debittering

## Abstract

Naringinase is an enzyme complex which exhibits α-l-rhamnosidase and β-d-glucosidase activity. This enzymatic complex catalyzes the hydrolysis of naringin (4′,5,7-trihydroxy flavanone 7-rhamnoglucoside), the main bittering component in grapefruit. Reduction of the level of this substance during the processing of juice has been the focus of many studies. The aim of the study was the immobilization of naringinase on chitosan microspheres activated with glutaraldehyde and, finally, the use of such immobilized enzyme for debittering grapefruit juice. The effect of naringinase concentration and characterization of the immobilized enzyme compared to the soluble enzyme were investigated. The maximum activity was observed at optimum pH 4.0 for both free and immobilized naringinase. However, the optimum temperature was shifted from 70 to 40 °C upon immobilization. The K_M_ value of the immobilized naringinase was higher than that of soluble naringinase. The immobilization did not change the thermal stability of the enzyme. The immobilized naringinase had good operational stability. This preparation retained 88.1 ± 2.8% of its initial activity after ten runs of naringin hydrolysis from fresh grapefruit juice. The results indicate that naringinase immobilized on chitosan has potential applicability for debittering and improving the sensory properties of grapefruit juices.

## 1. Introduction

Naringin, a flavanone diglycoside, is the main component responsible for the bitter taste of citrus fruits. Based on the available scientific publications, the concentration of naringin in commercial and fresh grapefruit juice ranges from 104 to 867 µg mL^−1^ (0.179–1.493 mM) [1,2,3,4,5,6] and depends on grapefruit variety, grapefruit maternity, and juice extraction method. Although the concentration of naringin in grapefruit juice is low, it imparts a perceptible bitter taste to consumers. The bitterness of citrus fruits, mainly of grapefruit, is an undesirable quality for the juices industry [7]. Bitter taste is a major factor in determining the choice of a food product [8].

The naringin level can be reduced by different methods such as adsorptive debittering [9] and β-cyclodextrin treatment [10]. However, because of various limitations of these debittering technologies, the enzymatic hydrolysis of naringin could be used as an alternative procedure. The debittering of citrus juice can be achieved by naringin hydrolysis using naringinase. Naringinase treatment is a promising method due to the fact that it enhances the organoleptic properties while retaining its health-promoting properties [11]. Naringinase is an enzyme complex, exerting α-l-rhamnosidase (EC 3.2.1.40) and β-d-glucosidase (EC 3.2.1.21) activities [12]. The ability of naringinase to hydrolyze many glycosides, e.g., naringin, hesperidin, quercetin, diosgene, and rutin, is the main application for that enzyme [3,13]. Moreover, due to the health-promoting properties of hydrolyzed products, it can be useful in pharmaceutical, cosmetic, and food applications [14]. In view of naringinase’s activity, it has valuable applications in food technology, such as debittering citrus fruit juices by the hydrolysis of naringin and enhancing wine and juice aroma by the release of free aromatic compounds from natural glycoside precursors [15,16,17,18,19]. Furthermore, naringinase can be commercially attractive in the pharmaceutical industry due to its role in biotransformation of steroids [19] and antibiotics [20].

Naringin can be hydrolyzed to rhamnose and prunin by α-l-rhamnosidase activity of naringinase. Afterwards, β-d-glucosidase catalyzes the hydrolysis of prunin to glucose and naringenin. Naringenin is a flavanone, and, in contrast to the closely related naringin, is tasteless. This unique reaction of naringinase can be used for debittering citrus juice, mainly of grapefruit, and to increase acceptance by consumers as well as the maintenance of health properties [1].

However, the use of free naringinase creates a number of practical problems, including the impossibility of reuse of the enzyme, sensitivity to environmental changes, and separation of the enzyme from the solutions. These problems can be solved using a variety of immobilization methods [21].

In recent years, various methods for the immobilization of enzymes, such as attachment to carriers by covalent, multicovalent, ionic binding, adsorption, or cross-linkage and entrapment (encapsulation), have been developed [22].

A major challenge in industrial biocatalysis is the development of stable biocatalysts. The solid support systems generally stabilize the structure of the enzymes and as compared to their free forms, immobilized enzymes are generally more stable with easier recovery and reuse [22]. The stability of an immobilized enzyme is highly dependent on many factors, e.g., the choice of support and immobilization strategy, the conditions under which the enzyme molecules were immobilized, the chemical and physical structure of the support and the nature of its interaction with the carrier, including the stability of the immobilized enzymes with respect to binding position and number of bonds [23].

In contrast to free naringinase, the immobilized enzyme can be used several times. Furthermore, it is easier to separate from the reaction and more resistant to environmental changes in pH, temperature, and ionic strength. Another advantage of immobilized naringinase is lowering the total production cost of the enzyme reaction [24,25]. Enzymes are relatively expensive; therefore, discarding them after a single use is not economical. Enzyme immobilization is only economically sustainable when the immobilized protein is very stable and can be used for many reaction cycles for the catalysis [26].

The use of immobilized enzymes in the food industry requires cheap immobilization procedures. Additionally, the supports must contain reactive groups and must be totally nontoxic [14]. The choice of the immobilization method depends on the application of naringinase. Numerous methods for achieving debittering of citrus fruit juice by hydrolysis of naringin using immobilized naringinase have been developed. Naringinase has been successfully immobilized on different types of supports, such as porous glass beads [25], chitin [27], celite [28], glutaraldehyde-coated woodchips [29], alginate beads [17,30,31], κ-carrageenan beads [1], polyvinyl alcohol [14], and other polymeric materials [32,33]. Moreover, naringinase from *Aspergillus aculeatus* has been immobilized onto magnetic Fe_3_O_4_ nanoparticles [34]. A method for obtaining nanomagnetic cross-linked enzyme aggregates of naringinase has also been developed [35,36]. The naringinase subunit α-l-rhamnosidase of *Aspergillus terreus* was covalently immobilized on three magnetic supports (Dacron-hydrazide, polysiloxane/polyvinyl alcohol (POS/PVA), and chitosan) [37].

Chitosan is a good support for immobilization of enzymes in the food industry because it is nontoxic, biodegradable to harmless products, useable in different forms, such as powders, flakes, and gels, and, most importantly, it has high protein affinity [24,38]. The chitosan support can be structurally modified by reaction between carbonyl groups of glutaraldehyde and amine groups of chitosan. In this manner, it can afford copolymer materials with improved adsorption properties [39]. Among the scientific publications on immobilization, enzyme immobilization using glutaraldehyde is one of the most commonly featured methods [29,38,40,41]. Glutaraldehyde is a versatile reagent in enzyme crosslinking and immobilization and has been used in many instances as a crosslinking agent between a functionalized support and an enzyme, as an activator of supports, and as a crosslinker of enzymes and supports [42].

In this case, the chitosan molecule chains having primary amino groups are activated with glutaraldehyde to form chitosan microspheres [38,42]. Glutaraldehyde reacts with the amine groups of the enzyme and chitosan through the formation of Schiff bases and Michael adduct [43]. Monsan [43] stated that it is simple to activate aminated supports with glutaraldehyde, having one or two glutaraldehyde molecules per the primary amino group of the matrix. However, the use of conditions such as pH over 8 and higher glutaraldehyde concentrations yields an uncontrolled reaction, leading to polymerization of glutaraldehyde in solution. By controlling the activation conditions, it may be possible to activate all the amino groups in a support [44,45].

An enzyme may be immobilized on activated glutaraldehyde supports by a covalent reaction and the molecules may be adsorbed. The adsorption may be mixed, ionic, or hydrophobic [42]. A support activated with glutaraldehyde may give three different kinds of interactions with a protein: hydrophobic, anionic exchange, and covalent. The activation degree of the support has an exponential effect on the immobilization reaction rate of proteins. The ionic adsorption is much faster that the direct covalent reaction between glutaraldehyde and enzyme, and this immobilization mechanism was the first way of immobilization for naringinase. The method of enzyme immobilization on the activated glutaraldehyde support can be altered by changing the immobilization conditions. Using a moderate ionic strength (100–250 mM of NaCl) and a long period of immobilization favored the covalent immobilization of most enzymes [42].

Based on this information, the naringinase from *Penicillium decumbens* was immobilized on chitosan microspheres, and the conditions for immobilization and characterization of the immobilized enzyme were studied. The immobilized naringinase was applied in this study with the purpose of debittering grapefruit juice.

## 2. Results and Discussion

### 2.1. Influence of Naringinase Concentration on Immobilization

**Figure 1:**
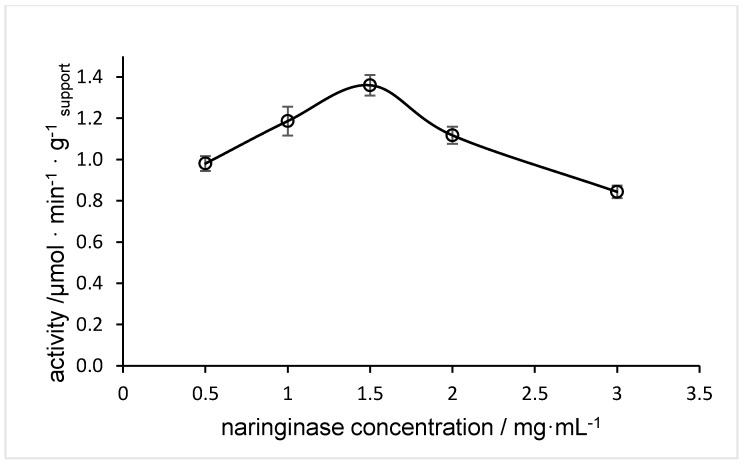
Effect of naringinase concentration on immobilization efficiency.

The relationship between the immobilized enzyme activity and the naringinase concentration used in the immobilization process is shown in Figure 1. An increase in the enzyme activity was observed with naringinase concentrations from 0.5 to 1.5 mg mL^−1^, and the activity of the immobilized enzyme reached a maximum (1.36 µmol min^−1^ g_support_^−1^) at a value of 1.5 mg mL^−1^.

Concentrations of naringinase above 1.5 mg mL^−1^ occurred with decreasing enzyme activity. This may be due to the fact, as reported by Doğaç and Teke [46], that the carrier concentration was not sufficient for immobilization. According to Guo et al. [47], this situation may result from a reduction of internal mass transfer and a decrease in the conformation transition of the enzyme, which may have resulted in lowered activity of the enzyme.

Other researchers, using different immobilization methods, have also found that the immobilization efficiency depends on the naringinase concentration [2,29,30,31,32,33]. Another possible association was noted by Nunes et al. [48], who reported that an increase in naringinase concentration tends to increase the overall enzyme activity.

The high efficiency observed with medium naringinase concentration led us to choose 1.5 mg mL^−1^ for subsequent studies.

### 2.2. Immobilization Yields

The activity immobilization yield (Ya) and the protein immobilization yield (Yp) were evaluated and are shown in Table 1.

Under the immobilization conditions, the activity of the naringinase immobilized on chitosan microspheres was found to be 1.36 µmol·min^−1^ ·g_support_^−1^, with an activity yield of 4.44% and a protein yield of 31.97%. The yield of immobilization by this method was significantly lower than yields of naringinase entrapment in gels [1,13,14].

### 2.3. Desorption of the Enzyme from the Support

The desorption course was monitored by measuring the immobilized enzyme activity before and after use of the desorbent, as seen in Table 2. The enzyme was released from the support; no activity was found on the support after incubation in 1 M NaCl solution.

According to Barbosa et al. [42], a support highly activated with glutaraldehyde may give three different kinds of interactions with a protein: hydrophobic, anionic exchange, and covalent. Using highly activated supports, in most cases, ionic exchange with the amino groups in the support is the first step in the immobilization of most enzymes. The nature of this noncovalent immobilization allows the process to be reversed by changing the ionic strength conditions [49].

In the case of the immobilization method used in the study, the short binding time of naringinase to the carrier activated with glutaraldehyde at low ionic strength favored the adsorption of the enzyme by means of ion exchange. The fact that the enzyme was completely eluted from the support by treatment of the immobilized enzyme preparation with 1 M NaCl solution confirms that the main mechanism of binding naringinase to chitosan activated with glutaraldehyde was ionic adsorption.

Ionic adsorption of naringinase on activated chitosan molecules is a simple and fast methodology for immobilizing the enzyme. After desorption of the used enzymes, the support can be reused to immobilize new batches of fresh naringinase. To prevent enzyme desorption, enzymes previously reversibly immobilized on a support can be cross-linked with the support (usually with glutaraldehyde or dextran aldehyde) [50,51].

### 2.4. Effect of pH on Naringinase Activity

**Figure 2:**
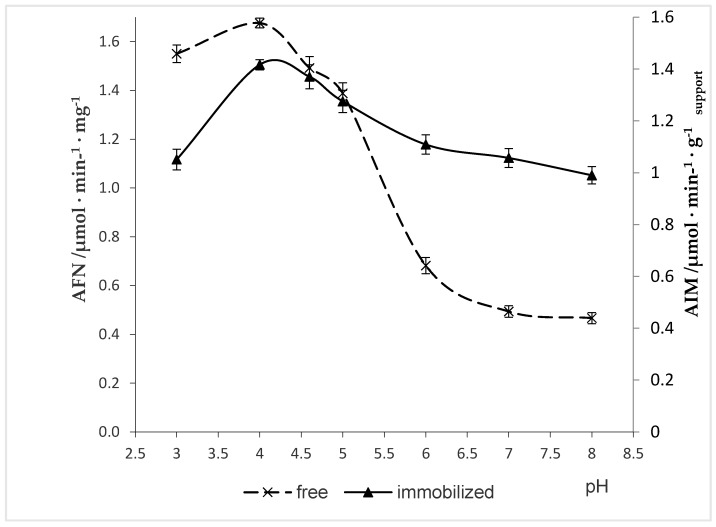
Effect of pH on activity of free and immobilized naringinase. AFN—activity of free naringinase; AIM—activity of immobilized naringinase.

The influence of enzyme immobilization on its activity at varying pH values was characterized. pH activity profiles were created by varying pH at a constant temperature (50 °C), as seen in Figure 2, as noted in the methods section.

The effect of pH on the activity of free and immobilized naringinase for naringin hydrolysis was examined from pH 3.0 to 8.0. Both the free and immobilized enzyme exhibited optimum activity at pH 4.0, which was similar to most fungal naringinases, which have maximum activity under acidic pH [52,53,54,55]. The activity of naringinase at pH 4.0 was 1.68 µmol·min^−1^·mg^−1^ and 1.41 µmol·min^−1^·g _support_^−1^ for free and immobilized naringinase, respectively. The pH profile of the immobilized naringinase revealed a broader profile of enzyme activity, which was suggestive of a protective role played by the chitosan microspheres and more noticeable at the higher pH values tested. Free naringinase presented a sharper activity profile than immobilized naringinase.

Mishra and Kar [55] also reported that immobilization makes the pH profile broader without shifting the pH optimum. Our results are in agreement with other reports, where immobilization of the naringinase does not affect the change of the optimal pH value (pH 4.0), namely in the study of Puri et al. [16] with alginate-entrapped naringinase from *Penicillium* sp., and of Nunes et al. [32], with naringinase from *Penicillium decumbens* immobilized in PVA (10%)–alginate beads. The constant pH value (4.5) for the free and immobilized enzyme was also reported in the work of Busto et al. [14] with naringinase from *Aspergillus niger* immobilized in PVA cryogel, but this pH value was slightly higher than that obtained in this study. However, a reduction of the optimal pH value by immobilization of the enzyme was recorded for the naringinase from *Penicillium decumbens* immobilized on celite—from pH 4.0 to 3.5 [28]. The lowering of optimum pH from 4.5 to 4.0 as a result of naringinase immobilization was also observed by Puri et al. [29] and Awad et al. [56]. In view of the fact that most fruit juices show acidic pH values, the low pH values are an important factor for the possible application of immobilized naringinase [56].

### 2.5. Effect of Temperature on Naringinase Activity

**Figure 3:**
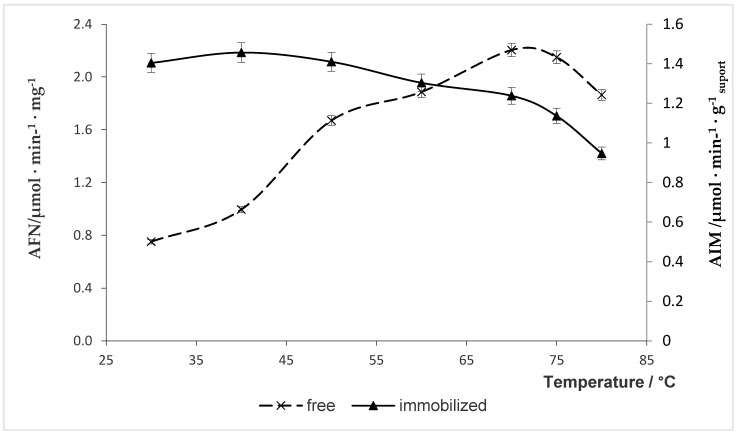
Effect of temperature on activity of free and immobilized naringinase. AFN—activity of free naringinase; AIM—activity of immobilized naringinase.

The effects of temperature on free and immobilized naringinase activities are shown in Figure 3.

The maximum activity was observed at 70 °C for free (2.20 µmol·min^−1^·mg^−1^) and 40 °C for immobilized (1.45 µmol·min^−1^·g _support_^−1^) naringinase. The immobilization of naringinase on the chitosan microspheres influenced the reduction of the optimum temperature of the enzyme.

Although it has been suggested that immobilized enzyme exhibits greater thermal stability [49], immobilization of naringinase on chitosan microspheres shifted the optimum temperature from 70 °C to 40 °C. The immobilization of naringinase on a chitosan carrier may cause a change in the conformation of the enzyme protein, which promotes better connection of the active center with the substrate. This change can be labile; therefore, at higher temperatures, the spatial structure is deformed, which results in reduced activity.

Şekeroğlu et al. [28] reported that immobilization of naringinase from *Penicillium decumbens* on celite by simple adsorption decreased the optimum temperature from 70 to 60 °C. These findings differ from the results reported by Puri et al. [16], who observed that the optimum temperature for naringinase entrapped in alginate was 55 °C and it was higher than for the free enzyme. Also, the immobilization of naringinase in the polyvinyl alcohol gel resulted in the increase of the optimal temperature from 60 to 70 °C relative to its free form [14]. A similar result was obtained by Awad [56], who immobilized naringinase by covalent binding into grafted gel beads. On the other hand, Nunes et al. [32] observed the optimum catalytic activity at 70 °C for both free and immobilized naringinase. The temperature profile of the immobilized enzyme was broader than that of free naringinase. Nunes et al. [32] also observed a broader temperature profile for the immobilized enzyme.

The optimal temperature of 70 °C for activity of free naringinase was consistent with the value measured by Şekeroğlu [28] and Nunes [32] for the enzymes from *Penicillium decumbens*. The optimal temperature values for the enzyme from *Aspergillus niger* are lower: 45 °C [55], 50 °C [17], and 60 °C [14].

The fact that the method used to immobilize naringinase does not increase the optimal temperature of enzyme activity does not constitute a disadvantage when using this enzyme to hydrolyze naringin of grapefruit juice. Running this process at a lower temperature protects the loss of thermolabile juice components (e.g., vitamin C, beta carotene) and helps maintain the sensory properties of the juice desired by consumers.

### 2.6. Thermal Stability of Free and Immobilized Naringinase

**Figure 4:**
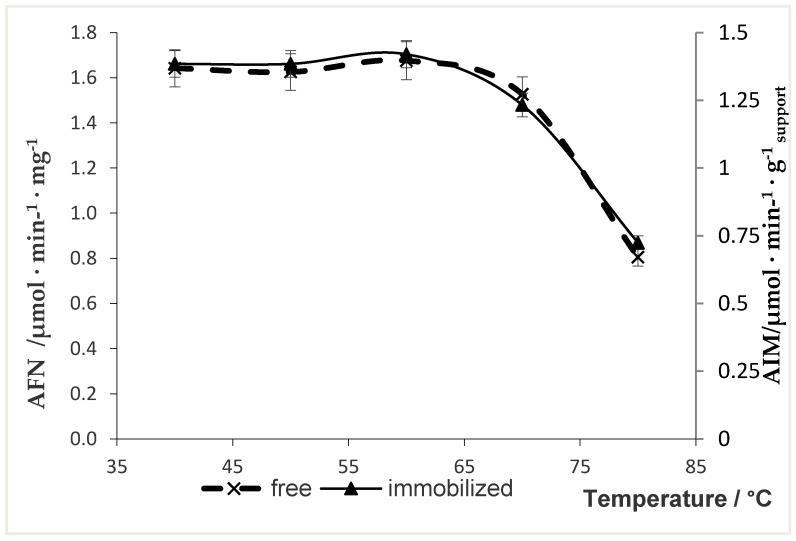
Thermal stability of free and immobilized naringinase. AFN—activity of free naringinase; AIM—activity of immobilized naringinase.

The thermal stability of the enzyme is of great importance for its application, especially in biocatalytic reactions. The enzyme immobilization on a support prevents its conformational changes under the influence of temperature, which results in increased durability of the enzyme against denaturation [57]. The results of thermal stability of the free and immobilized naringinase are shown in Figure 4.

The thermostability of the immobilized naringinase was studied between 40 and 80 °C and compared with the free enzyme. The chitosan-immobilized naringinase showed a similar performance to that of the free enzyme. Both enzymes were stable up to 60 °C and retained over 50% of the activity at 80 °C. At 80 °C, the activity of naringinase decreased from 1.85 to 0.99 µmol min^−1^ mg^−1^ and from 1.38 to 0.72 µmol in^−1^ g _support_^−1^ for free and immobilized enzyme, respectively. Soria et al. [37] obtained a similar course of thermal stability of free and immobilized α-l-rhamnosidase on ferromagnetic supports.

Immobilization of naringinase from *Penicillium decumbens* on mesoporous silica MCM-41 enhanced the thermal stability of the enzyme [58]. Puri et al. [29] reported that the immobilization of naringinase from *Penicillium* sp. on glutaraldehyde-coated woodchips caused a marked increase in the temperature stability of the enzyme. Moreover, as shown by Awad et al. [56], the immobilization of naringinase from *Aspergillus niger* into grafted gel beads highly improved the enzyme’s thermal stability.

The fact that the immobilization method used does not improve thermal stability does not represent a disadvantage when using this enzyme to hydrolyze naringin of grapefruit juice. The treatment of grapefruit juice based on the hydrolysis of naringin should be carried out at a relatively low temperature to prevent degradation of the thermolabile juice components.

### 2.7. Degree of Naringin Hydrolysis as Function of Time

**Figure 5:**
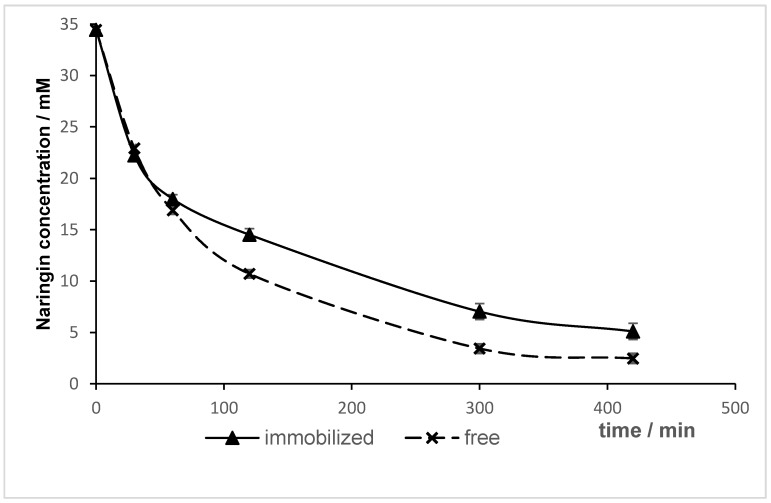
Residual concentration of naringin after hydrolysis as function of time.

The hydrolysis of naringin, using both soluble and immobilized enzyme, was investigated and the results are shown in Figure 5. A more rapid reaction was observed for free enzyme. After 30 min, a reduction of approximately 35% (from 34.45 mM to 22.23 mM) in naringin concentration was observed. A close result between free and immobilized enzymes was observed after 7 h; the free enzyme hydrolyzed 93% of naringin (from 34.45 mM to 2.47 mM) compared to 85% (from 34.45 mM to 5.10 mM) for the immobilized enzyme. Puri et al. [16] noted a higher rate of hydrolysis by the soluble naringinase (82% in 3 h) than by alginate-entrapped naringinase (72% in 3 h). Awad [56] obtained similar results as the free naringinase hydrolyzed more naringin (50% in 45 min) compared to the enzyme immobilized into grafted gel beads (40% in 45 min).

### 2.8. Determination of Kinetic Parameters

**Figure 6:**
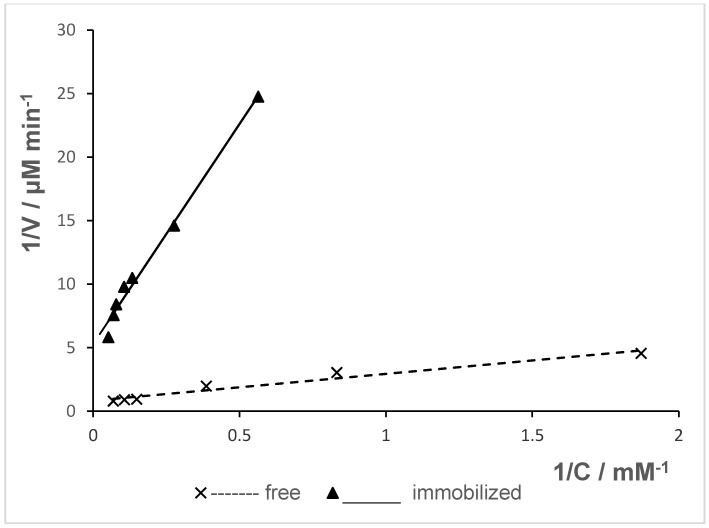
Lineweaver—Burk plots of free and immobilized naringinase. V—initial rate of naringin hydrolysis; C—naringin concentration.

The effect of naringin concentration on naringinase activity was investigated, and the results were calculated by linear regression of the Lineweaver–Burk plot, as shown in Figure 6.

The Michaelis constant (K_M_) and the maximum initial rate (V_max_) were evaluated for both free and immobilized naringinase and are shown in Table 3.

Immobilization of naringinase caused a decrease in the V_max_ value compared to the soluble enzyme. The apparent K_M_ value of immobilized naringinase was 2.5 times higher than that of free naringinase, which means the immobilized enzyme had lower affinity towards the substrate. In general, the increase in K_M_ value upon immobilizing naringinase has been reported by several authors [1,29,34]. A similar result—a two-fold increase in K_M_ value after immobilization of naringinase from *Penicillium decumbens*—was observed by Şekeroğlu et al. [28]. They reported that the values of K_M_ and V_max_ calculated were 1.22 mM and 0.45 μmol min^−1^ mg^−1^ for free naringinase and 2.16 mM and 0.3 μmol min^−1^ mg^−1^ for naringinase immobilized on celite, respectively. Puri et al. [16] also reported a higher K_M_ and lower V_max_ value for alginate-entrapped naringinase. However, the difference in the K_M_ value for free (8.4 mM) and immobilized (10 mM) enzyme was not as pronounced as in the discussed studies. The V_max_ values following the opposite trend of K_M_ suggests that the activity of the immobilized enzyme decreased in the course of binding [32].

Awad et al. [56] reported an increased K_M_ and V_max_ value for the immobilized enzyme, in contrast to our results. They obtained a K_M_ value of 5.9 mM and 41 mM for the free and covalent immobilized naringinase from *Aspergillus niger* and V_max_ from 8.3 mM min^−1^ for the free enzyme and 38.2 mM min^−1^ for the immobilized one. However, naringinase bound in mesoporous silica MCM-41 via adsorption with glutaraldehyde exhibited lower V_max_ and K_M_ values compared to the free enzyme [58]. Soraes and Hotkins [59] also reported that the apparent Michaelis constant for immobilized naringinase (K_M_ = 2.1 mM) was lower than that for the free enzyme (K_M_ = 3.6 mM).

As discussed in another paper, the increase of K_M_ may be caused by the steric limitations resulting from the support structure, the loss of enzyme flexibility necessary for substrate binding, or diffusion reduction of substrate and products [38].

### 2.9. Hydrolysis of Naringin from Grapefruit Juice

**Figure 7:**
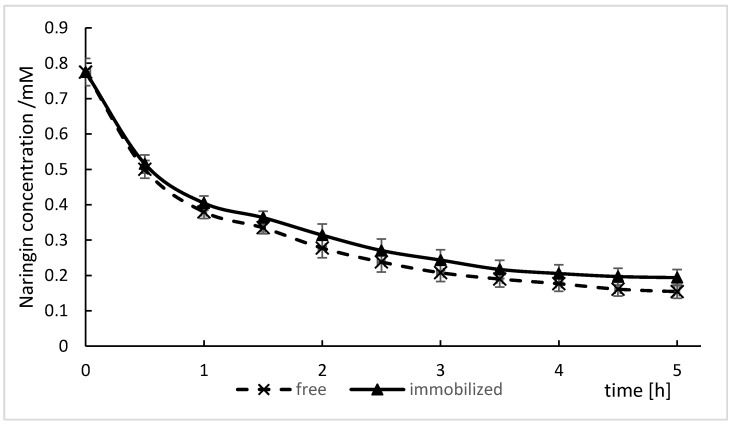
Hydrolysis of naringin from grapefruit juice as a function of time.

Naringin removal by the free and immobilized enzyme in fresh grapefruit juice was tested and the results are shown in Figure 7. It was observed that naringin was largely converted into naringenin by the free naringinase, whereas the enzyme immobilized onto chitosan resulted in hydrolysis of 75% of naringin after 5 h of enzymatic reaction.

This result is similar to that obtained for the hydrolysis of naringin in a standard solution, as seen in Figure 5. The initial content of naringin in the grapefruit juice was 450 μg mL^−1^ (0.775 mM). After 5 h, this amount was reduced to 112 μg mL^−1^ (0.193 mM), which, according to Soares and Hotchkins [60], means that the bitterness of the grapefruit juice was not perceptible.

Puri et al. [16] also observed a decrease in hydrolysis of naringin in kinnow mandarin juice (56% in 24 h) compared with naringin in standard solution (72% in 3 h) with application of alginate-entrapped naringinase. They suggested that the reduction of activity of the immobilized enzyme in the juice was a result of the inhibiting role of sugars and citric acid.

Mishra and Kar [55] used grapefruit juice as a substrate with naringinase entrapped in calcium alginate beads, thereby obtaining hydrolysis of 84% of naringin at 55 °C for 3 h. The treatment of grapefruit juice with naringinase immobilized in κ-carrageenan resulted in hydrolysis of 65% of naringin after 2 h [1]. Lei [58] demonstrated that the bitterness of grapefruit juice can be efficiently removed by naringinase. They observed that 96.09% and 95.03% of naringin was hydrolyzed by free and MCM41–naringinase, respectively. Naringinase immobilized on cellulose acetate nanofibers was applied to reduce the bitterness of grapefruit juice; as a result, 22.7% of naringin was converted to prunin [61]. A combined processing treatment of peeling and enzymatic hydrolyses using 5 U g^−1^ of pectinase and 0.4 U g^−1^ of naringinase for 60 min resulted in the decrease in pummelo juice of concentrations of naringin from 338 µg mL^−1^ to 42.4 µg mL^−1^ (from 0.582 mM to 0.73 mM) [62].

The reduction of naringin concentration in citrus juice by immobilized naringinase is possible. However, the difference in the results obtained may result from the use of various juices and concentrations of enzyme.

### 2.10. Operational Stability

**Figure 8:**
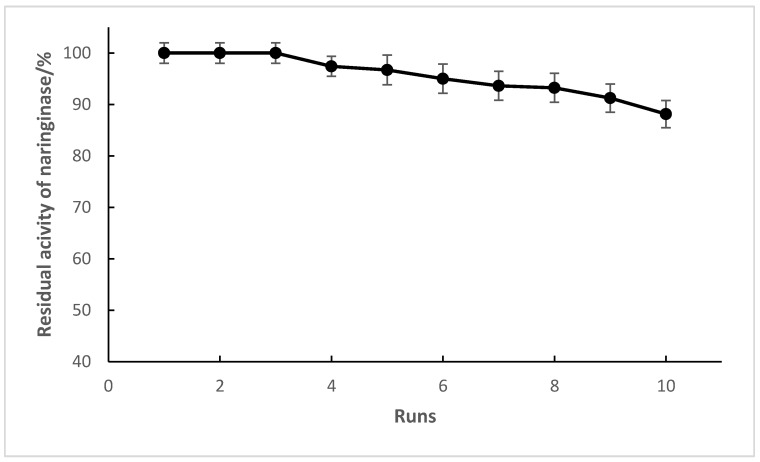
Degree of naringin hydrolysis contained in fresh grapefruit juice after 10 reutilization runs.

The operational stability of the immobilized naringinase was determined by repeating the process of naringin hydrolysis. The enzyme immobilized on chitosan microspheres was used ten times to hydrolyze naringin contained in fresh grapefruit juice. The results of this experiment are shown in Figure 8. The concentration of naringin as a result of the first run was reduced from 526 μg mL^−1^ (0.906 mM) to 328.67 μg mL^−1^ (0.566 mM), which means that 62.5% of naringin was hydrolyzed. The degree of hydrolysis after the first run was taken as 100%, and was used to compare with the results of subsequent runs. There was no loss of activity of the immobilized naringinase in the first three runs.

The immobilized naringinase retained 88.14 ± 2.77% of its initial activity after ten runs of naringin hydrolysis contained in fresh grapefruit juice. Slight losses of activity that were observed in the fourth and subsequent repetitions of the hydrolysis process could be due, among other causes, to mechanical interactions during mixing and then washing of the immobilized enzyme preparation.

The operational stability of the immobilized naringinase was satisfactory for debittering fruit juice and improving product quality. The operational stability of the naringinase immobilized on chitosan microspheres was higher when compared with results reported by Luo et al. [63] and Nunes [32], who used naringinase immobilized on porous silica material and entrapping it in PVA–alginate beads, respectively. Naringinase immobilized on silica material retained 61.81% of its initial enzymatic activity after being reused eight times. After eight runs, over 70% of the residual activity of naringinase immobilized in PVA–alginate beads was retained. Using a mixed method of adsorption/cross-linking with glutaraldehyde retained approximately 60% of enzyme activity after three cycles [28].

The operational stability of the enzyme immobilized on chitosan was only slightly lower compared to the results described by Puri et al. [29] and Vila-Real et al. [13]. Puri reported that naringinase immobilized by covalent binding to woodchips did not lose activity after seven operating cycles. Comparison of operational stability with the results of Vila Real et al. is difficult because this team did not use grapefruit juice as a substrate when testing operational stability.

## 3. Conclusions

The developed method of immobilization of naringinase on chitosan microspheres has provided an efficient and simple approach for the productive and reusable biocatalyst, together with ease in enzyme handling. The optimum activity over acidic pH and optimum low temperature of immobilized naringinase revealed that this enzyme can be suitable for application in acid environments, such as citrus juice, while maintaining the nutritional and organoleptic nature of the juice. Naringin, as the major bitter component of citrus juices, could be efficiently removed (75%) to a level below the limit of perceptibility by hydrolysis with naringinase immobilized on chitosan microspheres. This fact and the high operational stability of the naringinase immobilized on the chitosan support make this enzyme a promising candidate for future application of the enzyme. The main advantage of the presented method of immobilizing naringinase is its simplicity and short process time. It only takes 2 h to immobilize this enzyme. In addition, after regeneration of the carrier, by removing bound protein with a 1 M NaCl solution, it can be reused to immobilize the enzyme. Naringinase immobilized on chitosan microspheres also shows good operational stability, better than many other preparations obtained by other methods. These features indicate that immobilization of naringinase on chitosan macromolecules may find use in removing naringin from grapefruit juice. The lack of improvement in temperature stability is not a drawback in this case, because the processing of fruit juices should be carried out at lower temperatures to prevent loss of thermolabile ingredients and to preserve the sensory qualities of the product.

## 4. Materials and Methods

### 4.1. Materials

Naringinase from *Penicillium decumbens* (activity: 409 U g^−1^ solid) was obtained from Sigma–Aldrich (Poznań, Poland. Chitosan (medium molecular weight), naringin (assay 95%, HPLC), water, and acetonitrile (HPLC grade) were also purchased from Sigma–Aldrich. Glutaraldehyde (25%, by volume, aqueous solution) was from Merck (Warsaw, Poland). All other chemicals were of analytical grade and were obtained from various sources. Fresh grapefruits were bought in a local supermarket.

### 4.2. Preparation of Chitosan Microspheres

Preparation of chitosan microspheres followed the basic procedure described by Jiang [38], with minor modifications. Chitosan microspheres were prepared by applying reversed-phase suspension methodology, by using glutaraldehyde as a cross-linking reagent for the naringinase immobilization. Chitosan was dissolved in acetic acid (5%). 20 mL of dissolved chitosan (2.5% *m*/*v*) was added dropwise into 80 mL of paraffin containing 5 mL of emulsifier (Tween 80). The suspension was mechanically stirred for 30 min. After that 10 mL of 7.5% by volume glutaraldehyde as a cross-linking reagent was added to the microemulsion and stirred for 1 h at 40 °C. The whole mixture was adjusted to a pH of 9–10 by 4 M NaOH and incubated for 2 h at 70 °C. Finally, the product was filtered and washed successively with petroleum ether, acetone and distilled water. The microspheres were then dried for 12 h at 60 °C.

### 4.3. Immobilization of Naringinase

The conditions for immobilization followed the basic procedure described by Jiang et al. [38]. Before immobilization, 150 mg of chitosan microspheres were incubated in phosphate buffer (0.1 M, pH 7.0) for 20 h, and then the carriers were filtered. From 5 mg to 30 mg of naringinase in 10 mL of phosphate buffer (0.1 M, pH 7.0) was mixed with all chitosan microspheres for 2 h at 25 °C. After that, the immobilized naringinase was separated on a paper filter and next washed with phosphate buffer (0.01 M, pH 7) and distilled water.

### 4.4. Determination of Naringinase Activity

The naringin powder was dissolved in hot water at about 85 °C. A typical assay mixture comprised 1.5 mL of (34.45 mM) naringin, 1.5 mL of acetate buffer (0.1 M, pH 4.6), and 0.75 mL of a solution of naringinase (2.5 mg mL^−1^) or 150 mg of immobilized naringinase (support with the enzyme). The mixture was incubated in a water bath with constant shaking at 50 °C for 30 min. After that, the naringinase activity was determined by spectrophotometric determination of flavones (Davis method) [64] or reducing sugars (DNS method) [65]. The Davis method is based on the reactions of naringin with the alkaline diethylene glycol. 0.1 mL of this solutions and 0.1 mL of (4.0 M) NaOH were added to 4.8 mL of diethylene glycol (90%, *v*/*v*), and incubated for 10 min at room temperature. The intensity of the yellow color produced was measured at 420 nm. One unit of enzyme activity was defined as the amount of naringinase which hydrolyzed 1 μmol of naringin per minute under the conditions described.

All determinations were performed in triplicate. All values were expressed as mean ± standard deviation of the three replicate experiments.

### 4.5. Influence of Naringinase Concentration on Immobilization Efficiency

The effect of enzyme concentration on the activity of immobilized naringinase was evaluated. Five solutions of naringinase with concentrations ranging between 0.5 and 3 mg mL^−1^ were assayed.

### 4.6. Immobilization Yield

The activity immobilization yield, (Y_a_) was calculated from the following equation:

(1) $$Y_{a}=\frac{Xa}{Xa_{0}}\;\times 100\%$$

where X_a_ is the activity of immobilized enzyme per 1 g of support and X_a0_ is the total naringinase activity used for immobilization calculated per 1 g of support.

The protein immobilization yield, (Yp) was calculated from the following equation:

(2) $$Y_{p}=\frac{Xp}{Xp_{0}}\;\times 100\%$$

where Xp was the protein immobilized on the chitosan support and X_p0_ the total initial amount of protein. Xp was determined from the difference between the total amount added and that recovered after from the solution during the immobilization process and washings. Xp_0_ represents the total amount of protein used for immobilization.

The concentration of protein in the enzyme solution was determined before and after immobilization in the supernatant and the effluent of the washing steps. Protein concentration was determined using the Lowry method [66].

### 4.7. Desorption of Enzyme from the Support

The immobilized naringinase preparations were incubated with 10 mL of 1 M sodium chloride at 25 °C under shaking for 1 h. After this time, the support was separated from the solution by filtration and the residual activity of the preparation washed with 1 M NaCl was determined. All determinations were performed in triplicate.

### 4.8. Effect of pH and Temperature on Naringinase Activity

The effect of pH on activity of free and immobilized naringinase was monitored at 50 °C by using 0.1 M buffer solutions with pH ranging from 3.0 to 8.0. Enzyme assays were performed in 0.1 M McIlvaine buffer (pH 3.0–8.0).

The effect of temperature on activity of free and immobilized naringinase was measured at pH 4.0 (0.1 M acetate buffer), at temperatures between 40 and 80 °C. All determinations were performed in triplicate.

### 4.9. Thermal Stability of Naringinase

The thermal stability was assessed by measuring the activity of the enzyme exposed to different temperatures at pH 4.0 (0.1 M acetate buffer). Naringinase was preincubated for 30 min at temperatures ranging from 40 to 80 °C for the free and immobilized enzyme. After 30 min, the samples were cooled in water before the activity was determined under standard conditions. All determinations were performed in triplicate.

### 4.10. Determination of Kinetic Parameters

The effect of naringin concentration on enzyme activity of free and immobilized naringinase was studied. The kinetic constants, such as the maximum initial rate (V_max_) and the Michaelis constant (K_M_), were determined in the reaction medium (0.1 M acetate buffer pH 4.0 at 50 °C) by measuring rates of the reaction with substrate concentrations ranging from 0.5 mM to 20 mM or the free enzyme (1.7 mg·10 mL^−1^) and from 1 mM to 20 mM for the immobilized enzyme (2.5 mg·10 mL^−1^). For kinetic analysis, the initial reaction rate was determined. The initial reaction velocity, V_0_, was determined by drawing a straight line on the hydrolysis curve. The tangent of the curve was obtained based on differentiation of the polynomial equation as a function of the time course appearance of product data (the amount of hydrolyzed naringin). Microsoft Excel 2013 was used to perform the polynomial fitting. From the data, kinetic parameters were calculated using the Lineweaver–Burk method. All determinations were performed in triplicate.

### 4.11. Degree of Naringin Hydrolysis

The degree of naringin (34.45 mM) hydrolysis with both the free and immobilized enzyme as a function of time was followed. The hydrolysis was carried out at pH 4 (0.1 M acetate buffer) and at the temperature of 50 °C. The hydrolysis of the substrate was investigated at the start of the reaction and for another 7 h. Measurements were made every 30 min. All determinations were performed in triplicate.

### 4.12. Debittering of Grapefruit Juice by Naringinase

The hydrolysis of naringin was carried out in fresh grapefruit juice with free and immobilized naringinase. A total of 27.5 mL of freshly squeezed grapefruit juice and 1.38 mL of free enzyme (1 mg mL^−1^) or 800 mg of the immobilized enzyme were mixed in a conical flask and were incubated at 40 °C. The concentrations of naringin were determined in the juice before hydrolysis as well as after 30, 60, 120, 300, and 420 min of its duration. After treatment of the free enzyme, the reaction was stopped in boiling water for 5 min whereas samples with the immobilized enzyme were centrifuged. All samples were filtered through Whatman filters. The naringin level in grapefruit juice was analyzed by high performance liquid chromatography (HPLC). Separation was performed on a C-18 analytical column, at 280 nm at a 0.5 mL min^−1^ flow rate, and at room temperature. The injection volume was 20 µL. The mobile phase was composed of water and acetonitrile (23:77 by volume). All determinations were performed in triplicate.

### 4.13. Operational Stability

The operational stability of the immobilized naringinase was determined by repeating the process of naringin hydrolysis ten times from a fresh portion of grapefruit juice by the same immobilized naringinase preparation. For this purpose, 300 mg of the immobilized enzyme preparation was added to 5 mL of freshly squeezed grapefruit juice containing 526 μg mL^−1^ (0.906 mM) and mixed in a conical flask for 60 min at 40 °C. After this time, the immobilized enzyme preparation was separated from the juice by filtration, washed with 10 mL of distilled water, and a new batch of grapefruit juice was added. The content of naringin in the juice after hydrolysis was determined by HPLC as described in Section 4.12. The degree of naringin hydrolysis obtained as a result of the first cycle was taken as 100% of the activity of the immobilized naringinase. All determinations were carried out in triplicate.

## Figures and Tables

**Table 1:** Immobilization yields of naringinase on chitosan microspheres.

Offered Activity per Gram of Support (µmol min^−1^)	Activity of Immobilized Enzyme Per Gram of Support (µmol min^−1^)	Ya (%)	Offered Protein Per Gram of Support (mg)	Bound Protein Per Gram of Support [mg]	Yp (%)
30.6	1.36 ± 0.124	4.44 ± 0.048	75	23.98 ± 0.092	31.97 ± 0.32

Ya—activity immobilization yield; Yp—protein immobilization yield

**Table 2:** Activity of naringinase immobilized on chitosan microsphere before and after treatment with 1 M NaCl.

Desorption Method	Before Desorption	After Desorption	
	Activity [µmol·min^−1^·g_support_^−1^]	Activity [µmol·min^−1^·g_support_^−1^]	% Initial Activity
1 M NaCl	1.36 ± 0.124	0.000	0

**Table 3:** Kinetic parameters of free and immobilized naringinase.

	Vmax [μM min^−1^]	KM [mM]
Free naringinase	1.21	2.56
Immobilized naringinase	0.19	6.59
